# Association between 25-OH Vitamin D Deficiency and COVID-19 Severity in Pregnant Women

**DOI:** 10.3390/ijms232315188

**Published:** 2022-12-02

**Authors:** Johana Vásquez-Procopio, Johnatan Torres-Torres, Hector Borboa-Olivares, Salvador Espino Y Sosa, Raigam Jafet Martínez-Portilla, Mario Solis-Paredes, Mari-Cruz Tolentino-Dolores, Addy Cecilia Helguera-Repetto, Karla Cecilia Marrufo-Gallegos, Fanis Missirlis, Otilia Perichart-Perera, Guadalupe Estrada-Gutierrez

**Affiliations:** 1 Department of Immunobiochemistry, Instituto Nacional de Perinatología, Mexico City 11000, Mexico; 2 Department of Physiology, Biophysics and Neuroscience, Center for Research and Advanced Studies (Cinvestav), Mexico City 07360, Mexico; 3 Clinical Research Division, Instituto Nacional de Perinatología, Mexico City 11000, Mexico; 4 Community Interventions Research Branch, Instituto Nacional de Perinatología, Mexico City 11000, Mexico; 5 Coordination of Nutrition and Bioprogramming, Instituto Nacional de Perinatología, Mexico City 11000, Mexico; 6 Department of Gynecology and Obstetrics, Hospital General de México Dr. Eduardo Liceaga, Mexico City 06720, Mexico; 7 Research Division, Instituto Nacional de Perinatología, Mexico City 11000, Mexico

**Keywords:** immune system, pregnancy, asymptomatic, SARS-CoV-2, vitamin D deficiency

## Abstract

Evidence from studies in the general population suggests an association between vitamin D insufficiency/deficiency and COVID-19 susceptibility and disease severity. The present study was performed on 165 third-trimester pregnant women at the time of delivery. Seventy-nine women tested negative for SARS-CoV-2. From 86 women testing positive, 32 were asymptomatic, 44 presented a mild form of the disease, and 10 experienced severe symptoms. Serum 25-OH vitamin D levels were measured on blood samples collected on admission. Low vitamin D levels were detected in symptomatic but not asymptomatic COVID-19 patients compared to healthy women (*p* = 0.0227). In addition, 20 (45.4%) pregnant women in the mild COVID-19 group and 6 (60%) in the severe group were vitamin D deficient (*p* = 0.030). On the other hand, lasso regression analysis showed that 25-OH vitamin D deficiency is an independent predictor of severe COVID-19 with an odds ratio (OR) of 5.81 (95% CI: 1.108–30.541; *p* = 0.037). These results show the relationship between vitamin D deficiency in pregnant women and the severity of COVID-19 infection and support the recommendation to supplement with vitamin D to avoid worse COVID-19 outcomes during pregnancy.

## 1. Introduction

COVID-19 caused by SARS-CoV-2 has affected all population groups, including children, the elderly, and pregnant women [1,2,3]. Pregnancy is a particular condition that has significant effects on the biological systems of a woman’s body [4]. Pregnant women present changes in the immune system; therefore, they are generally considered vulnerable to infectious diseases [5,6]. Several studies have reported that pregnant women who experienced severe symptoms of COVID-19 were at higher risk for cesarean delivery, postpartum hemorrhage, hypertensive disorders of pregnancy, preterm birth, and maternal death [7,8,9,10,11].

Vitamin D is a liposoluble vitamin and hormone obtained from diet and skin exposure to sunlight [12]. The classic functions attributed to vitamin D are related to calcium and phosphate homeostasis, acting in the intestine, kidneys, and bone [13], but research in recent years has revealed a diverse range of biological actions [14]. Vitamin D is essential for cell differentiation while inhibiting cell growth and modulating inflammatory and immune responses [15]. Vitamin D deficiency (below 50 nM/L (20 ng/mL)) [16] is a global public health problem that commonly affects the elderly and those with comorbidities such as obesity, diabetes, hypertension, respiratory disorders, recurrent infections, immune deficiency, and malignancies, as well as ethnic minorities living in temperate countries [17,18]. Interestingly, the same groups known to be at risk of vitamin D deficiency were amongst the worst affected by COVID-19 [19,20,21].

Given the broad spectrum of activity of vitamin D, its deficiency is involved in many pathologies [22,23]. Prior to the COVID-19 pandemic, a meta-analysis found a significant association between low serum vitamin D levels and the severity of acute respiratory tract infections [24]. Currently, a relationship between vitamin D deficiency and the risk of SARS-CoV-2 infection or severe COVID-19 has been established in epidemiological studies [25,26,27,28,29,30]. Low vitamin D concentrations might predispose patients with COVID-19 to severe outcomes not only via the associated hyperinflammatory syndrome but also by worsening pre-existing impaired glucose metabolism and cardiovascular diseases [31,32,33]. However, some studies have not found these associations [34]. An observational study of 410 Indian patients hospitalized for COVID-19 showed a high prevalence of vitamin D deficiency but no association between serum vitamin D levels and clinical outcomes of COVID-19 [35]. In addition, a retrospective study from the UK Biobank showed that both circulating 25-OH vitamin D concentrations and vitamin D deficiency were not associated with the risk of COVID-19 [36,37]. In a retrospective case-control study, 82.2% of hospitalized patients with COVID-19 had vitamin D deficiency, but no relationship was found between serum 25-OH vitamin D concentrations or vitamin D deficiency and severe outcomes [38]. Therefore, the role of low vitamin D status in severe COVID-19 is debatable and may vary according to age, region, and ethnicity [27].

Vitamin D deficiency has several effects during pregnancy, such as failure of placental implantation [39], impaired angiogenesis [40], disturbances of the immune system [41], oxidative stress [42], and high incidence of gestational diabetes, cesarean section, and preterm birth. In infants, the related outcomes are low birth weight [43], lower bone mass [44], and, possibly, bronchiolitis, asthma [45], type 1 diabetes [46], multiple sclerosis, and autism [47]. Furthermore, maternal vitamin D deficiency represents a public health issue and is considered the least diagnosed and treated nutrition deficiency worldwide [48,49]. In a recent cohort study, we showed that the prevalence of vitamin D deficiency (<20 ng/mL) and insufficiency (20 ng/mL–30 ng/mL) was high in Mexican pregnant women [50].

Despite rapid advances in the development of evidence for managing COVID-19, further data collection on the effects of COVID-19 during pregnancy is needed. Lower vitamin D concentrations in pregnant women with COVID-19 have been reported [51,52,53]. However, the association is still controversial, and the clinical severity of the infection may not associate with vitamin D status in pregnant women [54,55]. This study aimed to investigate the association between vitamin D deficiency with COVID-19 severity in third-trimester pregnant Mexican women.

## 2. Results

### 2.1. Description of the Cohort and Characteristics of the Study Population

A total of 165 pregnant women in the third trimester were included in the analysis. Eighty-six (52%) tested positive for SARS-CoV-2 by RT-qPCR, and seventy-nine (48%) were negative. Amongst the positive, 32 (19%) were asymptomatic, 44 (27%) had mild symptoms, and 10 (6%) developed severe COVID-19. The demographic characteristics of healthy and infected pregnant women are summarized in Table 1 and fully available in Table S1. There were significant differences in the gestational age at hospital admission (*p* = 0.0001), serum magnesium (*p* < 0.0001), and serum calcium (*p* = 0.005) between groups.

### 2.2. Vitamin D Status According to COVID-19 Categories

**Figure 1:**
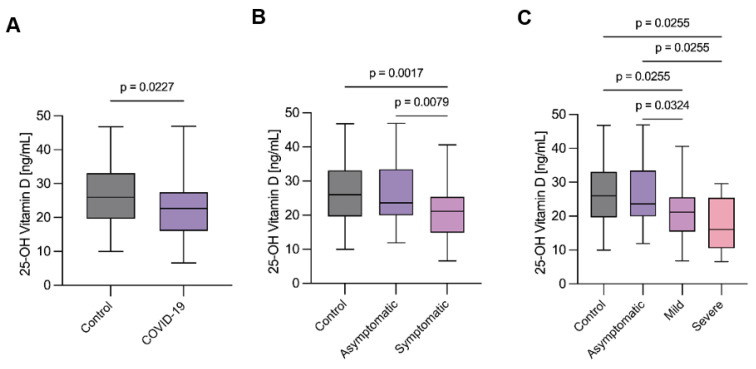
Serum 25-OH vitamin D levels in COVID-19 and control women in the third trimester of pregnancy. (**A**) 25-OH vitamin D levels in healthy pregnant women and COVID-19 group. (**B**) 25-OH vitamin D concentrations between asymptomatic and symptomatic group compared with control. (**C**) 25-OH vitamin D levels according to symptomatology. Control = 79; Asymptomatic = 32; Symptomatic = 54; Mild = 44; Severe =10. Standard deviations of the mean are shown in all panels. Comparisons between groups were performed using one-way ANOVA. Significance *p* < 0.05.

All COVID-19 positive pregnant women (asymptomatic and symptomatic) showed a significantly decreased concentration of serum 25-OH vitamin D compared with controls (*p* = 0.027) (Figure 1A). Serum vitamin D concentrations were significantly lower in symptomatic COVID-19 pregnant women compared to the asymptomatic group (*p* = 0.0079) and healthy pregnant controls (*p* = 0.0017) (Figure 1B). In contrast, no significant differences were found between asymptomatic COVID-19 pregnant women and healthy pregnant controls. Additionally, we observed that the patients with severe symptoms also showed statistically lower vitamin D concentration compared with the control group (*p* = 0.0255) (Figure 1C).

### 2.3. Distribution of the Patients According to 25-OH Vitamin D Levels

A statistically significant difference was found when we stratified results by vitamin D concentration in terms of deficiency (<20 ng/mL), insufficiency (20–30 ng/mL), and sufficiency (≥30 ng/mL). Twenty (45.4%) pregnant women in the mild COVID-19 group and six (60%) in the severe group were deficient in vitamin D (*p* = 0.030) (Table 2).

### 2.4. Association between 25-OH Vitamin D and COVID-19 Severity

We performed multivariate regression, including maternal age, gestational age, pregestational body mass index (BMI), diabetes, hypertension, gestational diabetes, magnesium, calcium, and vitamin D levels, using a cut-off value < 20 ng/mL considering the presence or absence of 25-OH vitamin D deficiency. The independent predictor of severe COVID-19 was 25-OH vitamin D deficiency showing an odds ratio (OR) of 5.81 (95% CI: 1.108–30.541; *p* = 0.037; Table 3). For comparison, we fitted a prediction model via lasso regression. Selected variables obtained were 25-OH vitamin D deficiency, gestational age, cholesterol, and magnesium. Similar performance was obtained (Table 4), and the only statistically significant independent predictor of severe COVID-19 was 25-OH vitamin D deficiency, showing a coefficient of 12.51 (95%CI: 4.097–20.929; *p* = 0.004).

### 2.5. Performance of Lasso Regression Comparable to Traditional Statistical Models

Similar performance was obtained with a false positive rate (FPR) of 15% (Table 5), and the logistic model performance had an area under curve (AUC) of 0.911 and a detection rate (DR) of 0.70; the lasso model had an AUC of 0.895 and a DR of 0.667. The elastic net via lasso regression and the logistic regression reflects the association of 25-OH vitamin D deficiency with COVID-19 severity in women in the third-trimester of pregnancy (Table 5).

## 3. Discussion

Prior observational studies have either demonstrated an association between vitamin D deficiency and the severity/mortality of COVID-19 [25,28,29,30,31,33,56] or failed to support such an association among hospitalized COVID-19 patients [34,35,36,37,38,57,58]. Thus, low vitamin D status as a risk factor for severe COVID-19 remains controversial. On the other hand, COVID-19 impacts pregnant women and their neonates [7,8,10], but vitamin D during pregnancy with SARS-CoV-2 has been investigated in a few cases [51,52,53,54,55]. Here, we report that women infected with SARS-CoV-2 had lower vitamin D levels than healthy pregnant controls. Additionally, even lower levels were observed in women with severe symptoms, which is consistent with disease progression. Our findings in Mexico are in line with those of Turkish [51] and French [52] pregnant groups, using the same definition of vitamin D deficiency [16]. It is worth considering that vitamin D status varies widely with the country of residence, age, ethnicity, and adequate exposure to the sun.

Vitamin D deficiency is common among pregnant women [48,49]. A meta-analysis of observational studies has shown a positive association between low vitamin D levels and adverse pregnancy outcomes such as preeclampsia, gestational diabetes mellitus, preterm birth, and SGA (small for gestational age) [59]. In this report, vitamin D deficiency (<20 ng/dL) was frequent (60%) in the severe group of COVID-19 patients. Vitamin D deficiency is associated with increased autoimmunity and an increased susceptibility to infection [12,22,25]. It is thought that adequate vitamin D levels (>30 ng/dL) reduce the inflammatory response to SARS-CoV-2 by increasing anti-inflammatory cytokines such as interleukin-4 (IL-4) and IL-10 levels and decreasing the concentrations of pro-inflammatory cytokines such as IL-1β, tumor necrosis factor-α (TNF-α), IL-8, IL-12, and, especially, IL-6 [60,61]. In particular, vitamin D induces the conversion of monocytes to macrophages and influences the activity of dendritic, T, and B cells [62].

Although a low serum 25-OH vitamin D concentration has been reported in COVID-19 pregnant women, not all studies consider the form of the disease (asymptomatic, mild, severe) [55]. Here, using the cut-off value of 25-OH vitamin D deficiency (20 ng/dL), serum biomarkers, and anthropometric and maternal medical history data, we found that vitamin D deficiency was the only variable associated to the severity of COVID-19, even after adjusting for other significant variables such as calcium, magnesium, BMI, hypertension, diabetes, and maternal age. Other studies have shown risk factors that predict severe disease, including age, BMI, and pre-existing comorbidities [20,21], but these were not significant in our cohort. A second model was created using an elastic net, a regularized regression method that linearly combines the ridge and lasso regressions. Both models reflected the association of 25-OH vitamin D deficiency with COVID-19 severity in pregnant women. Thus, the present work describes that women in the third trimester of pregnancy infected with SARS-CoV-2 have low vitamin D levels and also describes their association with the severity of the disease.

Some randomized controlled trials have shown that vitamin D supplementation is beneficial for reducing infection but not for reducing intensive care unit admission or all-cause mortality in patients with moderate to severe COVID-19 [56,63]. Current evidence suggests that taking a vitamin D supplement to maintain a serum concentration of 25-OH vitamin D of at least 30 ng/mL (preferred range of 40–60 ng/mL) can help reduce the risk of COVID-19 and its severe outcomes, including mortality [64,65]. Further, in a meta-analysis of randomized trials with 49,419 participants, a protective effect of daily vitamin D supplementation compared to other regimens in preventing respiratory diseases was found (OR: 0.78; 0.65–0.94) [66]. Our results suggest that vitamin D supplementation in SARS-CoV-2 infection could have a role in reducing the risk of severe COVID-19 disease in pregnant women [51,53,63,64]. Even though vitamin D supplementation in pregnant women with SARS-CoV-2 infection has not been reported, it is relevant to promote adequate levels (>30 ng/mL) in these women. Moreover, vitamin D supplementation during pregnancy may reduce the risk of gestational diabetes, preeclampsia, preterm birth, and having low birthweight and small for gestational age newborns [67,68,69].

The main limitation of the research presented here is the low number of patients who developed the severe form of the disease, which was probably related to the course of COVID-19 being asymptomatic and mild in most pregnant patients. Furthermore, by analyzing pregnant women with COVID-19 infection at the time of delivery, we have not evaluated the possible impact of the infection during pregnancy. Another weakness is the cross-sectional design, which does not allow us to establish a causal relationship between predictors and outcomes. Due to the pandemic situation, no information about diet and exposure to the sun was collected in this study.

To summarize, we identified serum 25-OH vitamin D levels as a risk factor for severe COVID-19 in women in the third-trimester of pregnancy. Our results could be explained by the crucial role of vitamin D in regulating the immune system [70] and its relationship with the prevention of respiratory infections and local inflammation at the pulmonary level. In other respiratory viruses, such as respiratory syncytial virus and influenza, vitamin D plays a role in preventing disease and limiting disease severity [22,41,45].

## 4. Materials and Methods

### 4.1. Study Design

This is a retrospective study including 165 women in the third-trimester of pregnancy who delivered at the National Institute of Perinatology and the Hospital General de México with Dr. Eduardo Liceaga in Mexico City, Mexico between June and December 2020. All women were tested for SARS-CoV-2 infection at admission by reverse transcription polymerase chain reaction (RT-qPCR) from nasopharyngeal swabs at least 24–48 h before delivery. Women with a positive test were classified into asymptomatic (*n* = 32) and symptomatic (*n* = 54) groups based on the absence of symptoms or the presence of at least one of the symptoms consistent with COVID-19 [71,72]. For symptomatic patients, the severity of illness due to COVID-19 was assessed according to oxygen saturation level (SatO_2_), the need for an extended period of hospitalization (unrelated to the pregnancy), intensive care unit admission, mechanical ventilation, or maternal death [73,74]. Thus, 10 pregnant women were included in the severe group, while the remaining 44 were included in the mild group, characterized by minor symptoms. A healthy control group of women with a negative test was also evaluated (*n* = 79). Demographics, comorbidities, symptomatology, hospitalization status, and other clinical information were obtained from the medical records (Table S1). Twin pregnancy and HIV-positive tests were considered exclusion criteria.

### 4.2. Quantification of 25-OH Vitamin D

Blood samples were collected at delivery using BD Vacutainer^®^ Trace Element Serum Tubes. Blood was centrifuged at 3500 rpm for 5 min, and serum was aliquoted and stored at −80 °C until use. The quantification of serum 25-OH vitamin D was performed by ELISA (chemiluminescent immunoassay; Architect Abbott Diagnostics, Lake Forest, IL, USA). The adjustment curve was created by duplication with six points. An acceptable coefficient of variation was considered as < 5%. An insufficient status was considered when serum concentrations were < 30 ng/mL, and a deficient status was considered when concentrations were < 20 ng/mL

### 4.3. Statistical Analysis

Statistical analyses were performed using GraphPad Prism 9.1.2 and Stata Statistical Software Release 17 (College Station, TX: Stata Corp LLC). Continuous variables were expressed as the median and interquartile range (IQR), and categorical data were expressed as number and percentage and analyzed using the Chi-square or Fisher’s exact test. The Kolmogorov–Smirnov test was applied to evaluate normality. For normally distributed data, the one-way ANOVA test was used to compare between groups, and the Kruskal–Wallis test was used otherwise. The prediction model was developed using the cut-off value of 25-OH vitamin D deficiency. For model construction, we used a nested logistic regression adding anthropometric variables and serum biomarkers to a model integrated by maternal medical history variables using forward and backward stepwise logistic regression analysis in which a *p*-value ≤ 0.05 was used for model-inclusion and a *p*-value > 0.05 was the criteria for model elimination. The second model was created by an elastic net. The elastic net typically outperforms classic regression methods by performing both shrinkage and automatic selection of predictors [75]. Due to the automatic selection of predictors achieved by penalty, no previous subset selection must be performed, thereby reducing the variance and instability of the prediction model; this is an advantage over previous methods [76]. Automatic selection of predictors performed in elastic net results in a simpler, sparse model that includes only a subset of variables, thereby allowing for better model interpretation [75,76]. Ten-fold cross-validation was used to determine the shrinkage parameter of the elastic net. The performance of the models created was assessed at 10% and 15% false positive rates (FPR) and compared using the area under the curve (AUC).

## 5. Conclusions

Our results show a relationship between vitamin D deficiency and COVID-19 infection in women in the third trimester of pregnancy. We found that decreased vitamin D levels may be a risk factor for developing severe SARS-CoV-2 infection during pregnancy. Additionally, this study may support the use of a supplement with vitamin D to avoid worse COVID-19 outcomes during pregnancy. However, more research is needed in this important field.

## Figures and Tables

**Table 1:** Demographic characteristics of patients.

Variable	COVID-19 Negative (*n* = 79)	COVID-19 Positive			
		Asymptomatic (*n* = 32)	Mild (*n* = 44)	Severe (*n* = 10)	*p*-Value
Age, median (IQR), years	29 (25–33)	31 (24–37)	30 (27–34)	31 (28–36)	0.209
Gestational week, median (IQR)	38.3 (38.0–38.5)	38.6 (38.0–39.3)	38 (35.8–39.0)	33.3 (28.4–36)	0.0001 ****
Pre-pregnancy BMI (kg/m^2^), mean ± SD	27.0 (24.0–30.0)	28.0 (23.0–32.5)	28.7 (24.8–31.1)	28.7 (22.5–35.4)	0.315
Pre-pregnancy obesity *n* (%)	19 (24%)	9 (28%)	21 (48%)	4 (40%)	0.053
Hypertension, *n* (%)	2 (3%)	3 (9%)	4 (9.09%)	1 (10%)	0.105
Diabetes, *n* (%)	1 (1%)	1 (3%)	0	0	0.556
Hypothyroidism, *n* (%)	16 (20%)	6 (18%)	6 (13%)	0	0.381
Preeclampsia, *n* (%)	11 (14%)	1 (3%)	4 (9%)	1 (10%)	0.371
Gestational diabetes, *n* (%)	8 (10%)	4 (12%)	6 (13%)	1 (10%)	0.670
25-OH Vitamin D, median (IQR), ng/mL	26 (19.6–33.1)	23.6 (19.9–32.8)	21.1 (15.4–25.4)	16.1 (11.0–25.2)	0.003 ***
Magnesium, median (IQR), mmol/L	0.50 (0.44–0.56)	0.60 (0.52–0.68)	0.66 (0.56–0.70)	0.63 (0.55–0.63)	0.0001 ****
Calcium, median (IQR), mmol/L	1.35 (1.26–1.44)	1.48 (1.37–1.61)	1.51 (1.39–1.63)	1.45 (1.34–1.58)	0.003 ***
Phosphorus, median (IQR), mmol/L	3.38 (2.95–3.80)	3.80 (3.22–4.20)	3.77 (3.00–4.50)	3.41 (3.00–4.02)	0.247

IQR: Interquartile range; BMI: Body mass index; SD: Standard deviation; *** *p* < 0.001, **** *p* < 0.0001.

**Table 2:** Distribution of the patients according to 25-OH vitamin D levels.

Group	COVID-19 Negative *n* (%)	Asymptomatic COVID-19 *n* (%)	Mild COVID-19 *n* (%)	Severe COVID-19 *n* (%)	*p*-Value
Sufficiency ≥ 30 ng/mL	30 (39%)	10 (31%)	6 (14%)	0	0.015
Insufficiency 20–30 ng/mL	29 (37%)	14 (44%)	18 (41%)	4 (40%)	
Deficiency < 20 ng/mL	20 (25%)	8 (25%)	20 (45%)	6 (60%)	

Chi-square test.

**Table 3:** Association between 25-OH vitamin D deficiency and severe COVID-19.

Severe COVID-19	OR	95% CI	*p*-Value
25-OH vitamin D deficiency	5.818	1.108–30.541	0.037
BMI	0.995	0.878–1.127	0.941
Hypertension	0.933	0.089–9.781	0.954
Gestational diabetes	1.189	0.111–12.694	0.886
Maternal age	1.02	0.905–1.149	0.739
Magnesium	0.102	0.002–36.830	0.448
Calcium	0.307	0.003–25.681	0.601

BMI: Body mass index; OR: Odds ratio; CI: Confidence interval.

**Table 4:** Comparation between regression model of 25-OH vitamin D deficiency as a predictor of severe COVID-19.

Regression Model	Coefficient	95% CI	*p*-Value	R2
Logistic	5.818	1.108–30.541	0.037	0.0818
Lasso	12.513	4.097–20.929	0.004	0.3289

OR: Odds ratio; CI: Confidence interval.

**Table 5:** Performance of 25-OH vitamin D for the prediction of severe COVID-19.

Regression Model	AUC 95% CI	DR at 10% FPR (95% CI)	DR at 15% FPR (95% CI)
Logistic model	0.911 (0.849–0.972)	0.687 (0.244–0.900)	0.7 (0.400–1.00)
Lasso model	0.895 (0.800–0.991)	0.444 (0.111–1.000)	0.667 (0.222–1.000)

AUC: Area under of curve; DR: Detection rate; FPR: False positive rate; CI: Confidence interval.

## Data Availability

The original contributions presented in the study are included in the article/Supplementary Material; further inquiries can be directed to the corresponding authors.

## Supplementary Materials

The following supporting information can be downloaded at: https://www.mdpi.com/article/10.3390/ijms232315188/s1. The data used to support the findings of this study have been provided as a Supplementary Excel File (Table S1).
